# Ferric carboxymaltose and SARS-CoV-2 vaccination-induced immunogenicity in kidney transplant recipients with iron deficiency: The COVAC-EFFECT randomized controlled trial

**DOI:** 10.3389/fimmu.2022.1017178

**Published:** 2023-01-04

**Authors:** Joanna Sophia J. Vinke, Dania H. A. Altulea, Michele F. Eisenga, Renate L. Jagersma, Tessa M. Niekolaas, Debbie van Baarle, Marieke van Der Heiden, Maurice Steenhuis, Theo Rispens, Wayel H. Abdulahad, Jan-Stephan F. Sanders, Martin H. De Borst

**Affiliations:** ^1^ Department of Nephrology, University Medical Center Groningen, Groningen, Netherlands; ^2^ Department of Immunology, University Medical Center Groningen, Groningen, Netherlands; ^3^ Department of Immunopathology, Sanquin Research, Amsterdam, Netherlands

**Keywords:** iron deficiency, SARS-CoV-2, kidney transplantation, vaccination, randomized controlled (clinical) trial

## Abstract

**Background:**

Kidney transplant recipients (KTRs) have an impaired immune response after vaccination against severe acute respiratory syndrome coronavirus-2 (SARS-CoV-2). Iron deficiency (ID) may adversely affect immunity and vaccine efficacy. We aimed to investigate whether ferric carboxymaltose (FCM) treatment improves humoral and cellular responses after SARS-CoV-2 vaccination in iron-deficient KTRs.

**Methods:**

We randomly assigned 48 iron-deficient KTRs to intravenous FCM (1-4 doses of 500mg with six-week intervals) or placebo. Co-primary endpoints were SARS-CoV-2-specific anti-Receptor Binding Domain (RBD) Immunoglobulin G (IgG) titers and T-lymphocyte reactivity against SARS-CoV-2 at four weeks after the second vaccination with mRNA-1273 or mRNA-BNT162b2.

**Results:**

At four weeks after the second vaccination, patients receiving FCM had higher plasma ferritin and transferrin saturation (*P*<0.001 vs. placebo) and iron (P=0.02). However, SARS-CoV-2-specific anti-RBD IgG titers (FCM: 66.51 [12.02-517.59] BAU/mL; placebo: 115.97 [68.86-974.67] BAU/mL, *P*=0.07) and SARS-CoV-2-specific T-lymphocyte activation (FCM: 93.3 [0.85-342.5] IFN-ɣ spots per 10^6^ peripheral blood mononuclear cells (PBMCs), placebo: 138.3 [0.0-391.7] IFN-ɣ spots per 10^6^ PBMCs, *P*=0.83) were not significantly different among both arms. After the third vaccination, SARS-CoV-2-specific anti-RBD IgG titers remained similar between treatment groups (P=0.99).

**Conclusions:**

Intravenous iron supplementation efficiently restored iron status but did not improve the humoral or cellular immune response against SARS-CoV-2 after three vaccinations.

## Introduction

Coronavirus Disease 2019 (COVID-19) has affected more than 500 million people worldwide since the beginning of the pandemic early 2020, leading to more than six million deaths (1). Kidney transplant recipients (KTRs) who are infected with severe acute respiratory syndrome coronavirus-2 (SARS-CoV-2) have an increased risk of adverse outcome, with a 17-23% mortality rate (2–5). Immunosuppressive medication in KTRs impedes powerful humoral (6–11) and cellular (10, 12) immune responses against SARS-CoV-2 after vaccination. Previous studies identified higher age, pre-transplantation dialysis, deceased donor type, worse graft function, recent use of high-dose corticosteroids and use of mycophenolic acid as risk factors for a poor antibody response after SARS-CoV-2 vaccination in KTRs (8, 10, 13). Still, identification of new modifiable risk factors for an impaired immune response in KTRs is urgently needed (6, 8, 10).

Iron deficiency (ID) is highly prevalent after kidney transplantation (14), and has recently been proposed as potential treatment target to improve vaccine efficacy (15). Iron is involved in nucleotide synthesis for replication of deoxyribonucleic acid and in mitochondrial energy metabolism (16). Therefore, rapidly proliferating cells such as lymphocytes are prone to be affected by ID. Recent studies demonstrated impaired B-cell proliferation, plasma cell differentiation and immunoglobulin production in iron-deficient mice (17). In humans, ID is associated with reduced antibody production in response to various vaccinations (17), while pre-vaccination iron supplements improved vaccination-induced immune responses (18). Given these observations, the European Hematology Association recently published an expert opinion advising to correct ID before vaccination against SARS-CoV-2 (19). Whether correcting ID improves SARS-CoV-2 vaccine efficacy in KTRs is unknown. Therefore, we aimed to address the hypothesis that in iron-deficient KTRs, ferric carboxymaltose (FCM) improves the humoral and cellular response after vaccination against SARS-CoV-2.

## Materials and methods

### Patient population and study design

COVAC-EFFECT is a secondary analysis performed in a subpopulation of the ongoing EFFECT-KTx study (NCT03769441, also covering COVAC-EFFECT), a randomized, placebo-controlled, parallel-arm clinical trial aiming to address the effects of FCM versus placebo on exercise tolerance, cardiac function and other clinical outcomes in iron-deficient KTRs. In the mother trial, 158 subjects receive up to four doses of FCM (containing 500 mg Fe3+ per dose) or placebo (0.9% sodium chloride solution) intravenously, with six-week intervals. In case of severe hypophosphatemia (plasma phosphate ≤1.55 mg/dL) or active systemic infection on the day of the study visit, one treatment is withheld. In case of imminent iron overload, defined as a plasma ferritin level of ≥800 ug/L or 500-799 ug/L in combination with a transferrin saturation (TSAT) of ≥45% on the day of the study visit, patients in the FCM arm receive a dose of placebo instead of FCM. The study protocol of the EFFECT-KTx study and COVAC-EFFECT was approved by the medical ethical committee of the University Medical Center Groningen (METc 2018/482), conducted in accordance with the principles of the Declaration of Helsinki and consistent with the Good Clinical Practice guidelines provided by the International Council for Harmonization of Technical Requirements for Pharmaceuticals for Human Use. All participants had given their informed consent prior to enrollment in the EFFECT-KTx study as well as to enrollment in COVAC.

**Figure 1:**
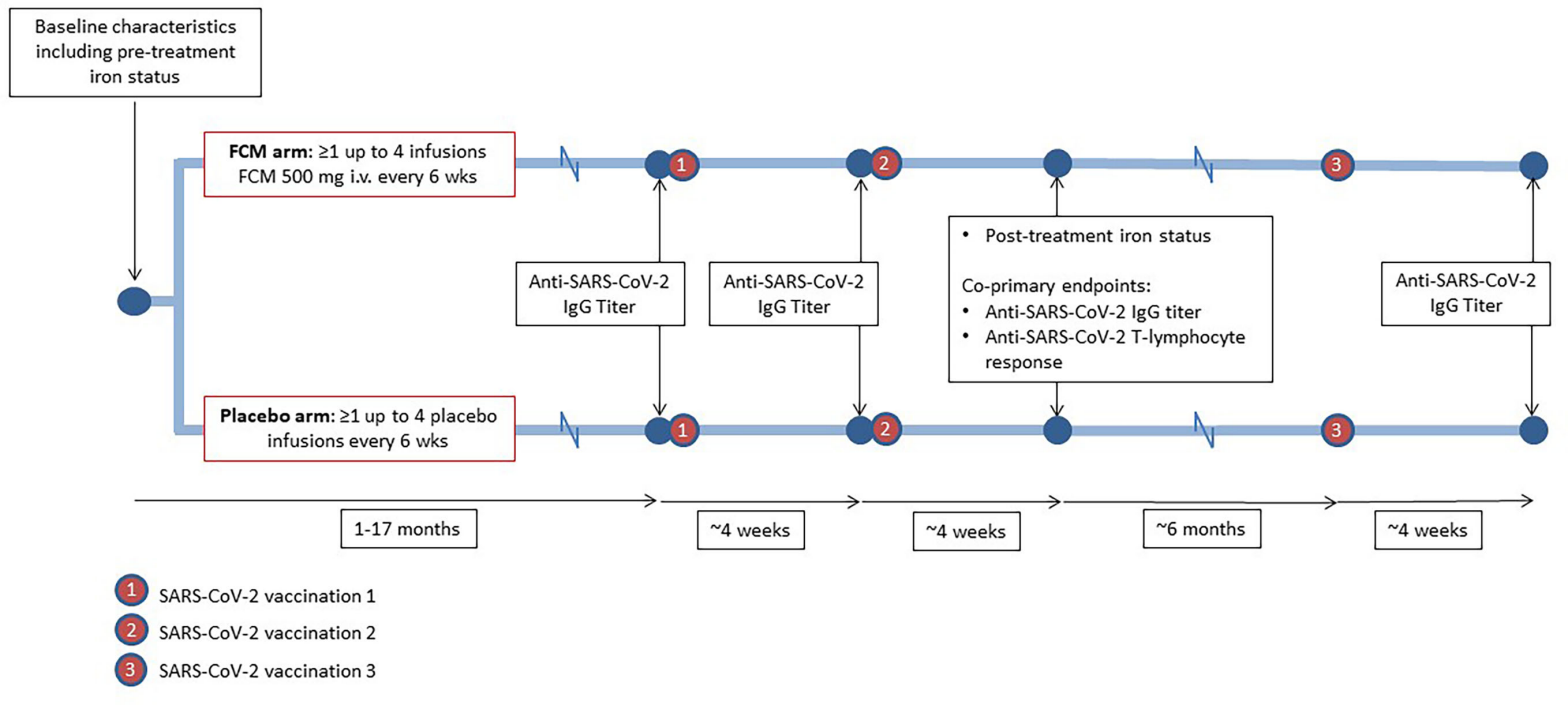
Study design. During participation in the EFFECT-KTx study, iron-deficient kidney transplant recipients are being randomized to receive four doses of ferric carboxymaltose (FCM) or placebo with six-week intervals after baseline measurements, including iron status assessment, have been performed. During or after participation in the EFFECT-KTx study, patients received three vaccinations against SARS-CoV-2. Before the first vaccination and four weeks after the successive vaccinations, anti-SARS-CoV-2 IgG titer was measured. Four weeks after the second vaccination, anti-SARS-CoV-2 T-lymphocyte response was assessed.

The current study could be performed since the ongoing EFFECT-KTx trial coincided with the Dutch national SARS-CoV2 vaccination program. Given this setting, a formal *a priori* sample size calculation was not performed; instead, all patients enrolled in the mother trial (EFFECT-KTx) who met the inclusion criteria for the current substudy were invited to participate. Participants who had received at least one treatment with study medication were eligible. The unblinded researchers who analyzed the data for the current study worked completely independent of the study team working on the mother trial, to ensure that these investigators remained blinded. An overview of the study design is provided in **Figure 1**. For the current study, we enrolled 48 iron-deficient EFFECT-KTx participants with a functional graft for more than six months post-transplantation who had not reported COVID-19 and who agreed to vaccination against SARS-CoV-2. Two patients who tested positive for SARS-CoV-2 within four weeks after the first or the second vaccination were excluded.

### Vaccination

Participants were vaccinated against SARS-Cov-2 as part of the Dutch national vaccination campaign. Patients who were eligible for early vaccination, because of high age or occupation in healthcare, received two mRNA-BNT162b2 vaccinations (COMIRNATY, ^®^Pfizer-BioNTech) with an interval of 35 days. All other participants received two mRNA-1273 vaccinations (^®^Moderna Biotech Spain, S.L.) with an interval of 28 days according to the manufacturer’s instructions. Six months after the second vaccination dose, participants received a third vaccination dose.

### Data collection and definitions

Co-primary outcomes of this study were the humoral and cellular immune responses at four weeks after the second vaccination. At this timepoint, SARS-CoV-2-specific anti-Receptor Binding Domain (RBD) immunoglobulin G (IgG) levels, expressed in international Binding Antibody Units (BAU)/mL, were measured in venous blood. Furthermore, capillary blood was collected with a self-collection finger prick set prior to the first and the second vaccination and four weeks after the third vaccination for additional SARS-CoV-2-specific anti-RBD IgG titer measurements. To assess cellular immunogenicity, peripheral blood mononuclear cells (PBMCs) were isolated from the blood sample that was taken four weeks after the second vaccination.

Baseline characteristics were registered and measurements were performed before treatment with FCM or placebo. Body Mass Index (BMI) was calculated as weight in kilograms divided by height in meters squared. Plasma creatinine was measured in venous blood with an enzymatic photometry assay (Roche Diagnostics, Mannheim, Germany). The estimated glomerular filtration rate (eGFR) was calculated using the Chronic Kidney Disease Epidemiology Collaboration (CKD‐EPI) equation, omitting the black race coefficient (20). Plasma iron was measured with a colorimetric assay, ferritin with an immunoassay and transferrin with a turbidimetric assay (Roche Diagnostics), all in venous blood samples. TSAT was calculated as 100 x (plasma iron (μg/dL) ÷ (total iron binding capacity (μg/dL)). Hemoglobin was measured in venous blood with spectrophotometry. ID was defined as a plasma ferritin concentration below 100 μg/L or a ferritin concentration between 100 and 299 μg/L in combination with a TSAT below 20% (21). Severe ID was defined as a ferritin concentration below 30 μg/L (22). IgG deficiency was defined as a total IgG concentration below 7.0 g/L (23).

### Anti-SARS-CoV-2 antibody response

Serum was isolated from capillary blood samples (fingerpick sampled at home) taken before the first and the second vaccination and four weeks after the third vaccination, or from venous blood sample drawn four weeks after the second vaccination. SARS-CoV-2-specific anti-RBD IgG antibodies were measured using an in-house (Sanquin, Amsterdam, the Netherlands) enzyme-linked immunosorbent assay (ELISA) assay, as described by Steenhuis et al. (24) IgG titers were compared with the World Health Organization reference sample (NIBSC code: 20/136) and expressed as BAU/ml. Cut-off for seroconversion was defined based on the 98^th^ percentile of signals of 240 pre‐outbreak plasma samples, corresponding to an anti-RBD IgG titer of ≥50 BAU/mL (24, 25). In addition, to detect previous exposure to SARS-CoV-2, a highly sensitive and specific total antibody SARS-CoV-2 RBD bridging assessment was performed on all samples, using a double antigen sandwich ELISA based assay developed by Sanquin, as described previously (24, 26). Outcome of this assay was compared to a Sanquin in-house calibrator of pooled convalescent plasma and expressed as normalized optical density units (nOD). An nOD >0.1 was considered as seropositive for total SARS-CoV-2- specific anti-RBD antibodies (24, 26). Furthermore, SARS-CoV-2-specific anti-nucleocapsid protein IgG antibodies were measured in samples from patients with a nOD >0.1 or an anti-RBD IgG titer of ≥50 BAU/mL at baseline, using an ELISA assay (26). Since these antibodies do not react to vaccination and are highly specific for previous exposure to the virus itself, they can be used to assess whether antibodies are induced by SARS-CoV-2 vaccination or infection.

### T-lymphocyte reactivity against Spike protein

In all participants, the SARS-CoV-2-specific T-lymphocyte response was measured after stimulation of PBMCs isolated from heparinized venous blood obtained four weeks after the second vaccination, using SepMate tubes (STEMCELL). The number of Interferon-gamma (IFN-ɣ)-producing T-lymphocytes after stimulation with SARS-CoV-2 Spike overlapping peptide pools was assessed using an IFN-ɣ enzyme-linked immune adsorbent spot (ELISpot) assay. SARS-CoV-2 S1 and S2 peptide pools (JPT Peptide Technologies), consisting of 15-mer peptides overlapping 11 amino acids that cover the entire sequence of the viral proteins were used for overnight stimulation of the PBMCs in a concentration of 0.5 µg/mL. 0.4% dimethyl sulfoxide (DMSO, Sigma) was used as negative control and Phytohaemagglutinin (Remel Europe Ltd; 4 µg/mL) as a positive control. Spot forming cells (SFC) were quantified with the AID ELISpot/Fluorospot reader and calculated to SFCs/10^6^ PBMCs. The average of the DMSO negative control was subtracted per stimulation. To define the total Spike-specific SFC, the SFC of the separate S1 and S2 peptide pools were summed. Results are expressed in number of IFN-ɣ spots per 10^6^ PBMCs. KTRs who had more than 50 IFN-ɣ spots per 10^6^ PBMCs were considered to be responders (27).

### Statistical analyses

We used IBM SPSS Statistics version 23.0 (SPSS Inc., Chicago, USA) and Prism 8.0 (GraphPad Software Inc., San Diego, USA) to analyze the data. Normally distributed data are presented as mean ± standard deviation (SD). Data with a skewed distribution are presented as median (interquartile range [IQR]). Categorical data are expressed as number (percentage).

To assess changes between baseline and post-vaccination measurements, we used the paired T-test for normally distributed data or the Wilcoxon-signed rank test for data with a skewed distribution. To assess differences between the two study arms at four weeks after the successive vaccinations, the independent samples T-test and Mann-Whitney Test were used. To adjust for differences in baseline lymphocyte count or eGFR, analysis of covariance was performed. Correlations were analyzed using the Spearman’s rank test.

The primary analyses were performed according to the intention-to-treat principle. We additionally performed ten sensitivity analyses. First, a per-protocol analysis was performed, excluding KTRs who were still iron-deficient after treatment with FCM, as well as KTRs who were not iron-deficient despite placebo treatment. In a second sensitivity analysis, we excluded four patients who were seropositive for total SARS-CoV-2-specific anti-RBD antibodies (n=3) or anti-SARS-CoV-2 IgG antibodies (n=1) at baseline. As a third sensitivity analysis, we excluded KTRs who were IgG-deficient at baseline. Fourth, we repeated the analyses in a subpopulation restricted to patients who had received the mRNA-1273 vaccine. In a fifth sensitivity analysis, only patients with severe ID (ferritin <30 μg/L) were included. Sixth, we analyzed patients on dual or triple immunosuppressive therapy separately. Seventh, patients who had received treatment with alemtuzumab, methylprednisolone or anti-thymocyte globulin <2 years prior to vaccination were excluded. In an eighth sensitivity analysis, the subgroup of patients who used mycophenolic acid was studied. Moreover, the subgroup of patients using mycophenolic acid with a dosage of 500mg twice daily was analyzed separately. Finally, we analyzed men and women separately. In all analyses, a P value of ≤0.05 was considered significant.

## Results

### Baseline characteristics and vaccines

Forty-six KTRs (age 53 [43-65] years, 61% male) were included. Baseline characteristics are shown in **Table 1**. Patient characteristics in both treatment arms were generally well balanced, except for small but significant differences in lymphocyte count, eGFR and prevalence of hypertension. Forty-one patients received the mRNA-1273 vaccine, and five KTRs received the mRNA-BNT162b2 vaccine. Four patients had a nOD of >0.1 or an anti-RBD IgG titer of ≥50 BAU/mL at baseline; three of them also had anti-nucleocapsid protein IgG antibodies at baseline. Time between enrollment in the EFFECT-KTx study and first vaccination was 33 ± 24 weeks.

**Table 1:** Baseline characteristics.

Characteristics	Total (N=46)	FCM (N=25)	Placebo (N=21)
Age, yr	53 (43–65)	55 (46–64)	51 (39-69)
Male sex, n (%)	28 (61)	12 (48)	16 (76)
Body mass index, kg/m^2^	26 ± 4	26 ± 4	27 ± 5
Diabetes, n (%)	13 (28)	6 (24)	7 (33)
Hypertension, n (%)	40 (87)	19 (76)	21 (100)
Alcohol consumption, units per week	0 (0-3)	0 (0-3)	1 (0–4)
Current tobacco use, n (%)	3 (7)	0 (0)	3 (14)
Time since transplantation, yr	3 (1-4)	2 (1–4)	3 (1–5)
History of dialysis, n (%)	27 (59)	16 (64)	11 (52)
Living donor, n (%)	34 (74)	17 (68)	17 (81)
Medication use			
Dual immunosuppressive therapy, n (%)	8 (17)	4 (16)	4 (19)
Triple immunosuppressive therapy, n (%)	40 (83)	21 (84)	17 (81)
Prednisone use, n (%)	46 (100)	25 (100)	21 (100)
Calcineurin inhibitor use, n (%)	42 (91)	23 (92)	19 (91)
Antiproliferative agent use, n (%)	40 (87)	21 (84)	19 (91)
Mycophenolic acid use, n (%)	38 (83)	20 (80)	18 (86)
Azathioprine use, n (%)	2 (4)	1 (4)	1 (5)
mTOR inhibitor use, n (%)	2 (4)	2 (8)	0 (100)
Anti-thymocyte globulin treatment ≤2 years, n (%)	1 (2)	1 (4)	0 (0)
Alemtuzumab treatment ≤2 years, n (%)	0 (0)	0 (0)	0 (0)
Methylprednisolone treatment ≤2 years, n (%)	0 (0)	0 (0)	0 (0)
Vaccination against SARS-CoV-2			
mRNA-1273 (Moderna), n (%)	41 (89)	23 (92)	18 (86)
mRNA-BNT162b2 (COMIRNATY), n (%)	5 (11)	2 (8)	3 (14)
**Time since last study treatment at time of first vaccination, weeks**	10 (2 – 39)	11 (2 – 42)	10 (2 – 38)
Laboratory parameters			
Hemoglobin, g/dL	13.4 ± 1.1	13.2 ± 1.1	13.5 ± 1.0
MCV, fL	90 ± 5	90 ± 7	89 ± 3
Leucocyte count - 10^9^/L	7.2 ± 1.8	7.0 ± 2.0	7.3 ± 1.6
**CRP, mg/L**	1.3 (0.7 – 3.0)	1.6 (0.9 – 2.9)	1.2 (0.6 – 3.6)
Lymphocyte count - 10^9^/L	1.5 ± 0.7	1.2 ± 0.5	1.7 ± 0.8
eGFR, mL/min/1.73 m^2^	63 ± 18	59 ± 16	68 ± 18
Iron, μg/dL	76.5 ± 24.0	75.4 ± 25.7	78.2 ± 22.9
Ferritin, μg/L	37 (26–70)	57 (29–79)	32 (24–62)
TSAT, %	21 ± 8	21 ± 8	21 ± 8
**TIBC, µg/dL**	373 ± 63	369 ± 70	378 ± 55
Total IgG, g/L	8.4 ± 2.0	8.3 ± 1.9	8.5 ± 2.2

Baseline characteristics, assessed on the morning before the first treatment with FCM or placebo, and vaccination characteristics. Data are presented as mean ± standard deviation (SD), median with interquartile range (IQR) or number (n) with percentage (%). CRP, C-reactive protein; eGFR, estimated glomerular filtration rate; FCM, ferric carboxymaltose; IgG, Immunoglobulin G; MCV; mean corpuscular volume; mTOR, mammalian target of rapamycin; SARS-CoV-2, severe acute respiratory syndrome coronavirus; TIBC, total iron binding capacity; TSAT, transferrin saturation.

### Iron status

**Figure 2:**
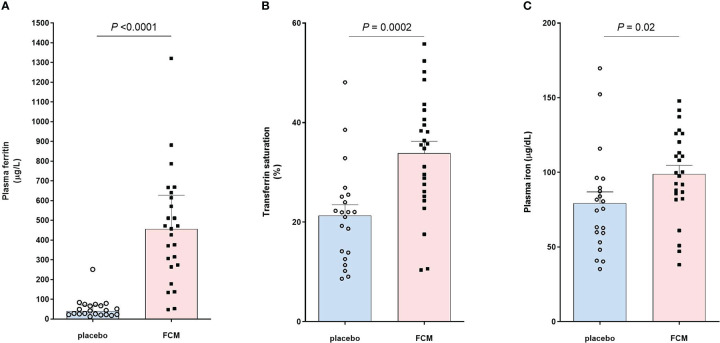
Effect of FCM or placebo on **(A)** plasma ferritin levels, **(B)** transferrin saturation and **(C)** plasma iron levels in iron-deficient KTRs.

Iron status was assessed at baseline before treatment with FCM or placebo and four weeks after the second vaccination, which was on average 42 ± 24 weeks after baseline (**Table 2**). Patients in the FCM arm showed an increase in plasma ferritin levels from 49 [26–79] μg/L to 464 [272–621] μg/L (*P*<0.001 vs baseline), while in the placebo group, ferritin did not significantly change (34 [24–62] μg/L to 42 [23–69] μg/L, *P*=0.39, **Figure 2A**). Ferritin levels at four weeks after the second vaccination were significantly higher in the FCM arm than in the placebo arm (*P*<0.001). TSAT also increased significantly in the FCM arm (from 21 ± 8% to 34 ± 12%, *P*<0.001), but not in the placebo arm (21 ± 8% vs 21 ± 10%, *P*=0.84 vs placebo baseline, *P*<0.001 vs FCM, **Figure 2B**). Plasma iron levels increased significantly in the FCM arm (from 75.4 ± 25.7 µg/dL to 98.8 ± 29.0 µg/dL, *P=*0.004), but not in the placebo arm (78.2 ± 22.9 µg/dL to 79.3 ± 34.1 µg/dL, *P*=0.89 vs placebo baseline, *P*=0.02 vs FCM, **Figure 2C**). Two months after the second vaccination, only two patients in the FCM arm were still iron-deficient. One patient was not iron-deficient anymore despite placebo treatment. Finally, there was a small but significant effect of FCM on hemoglobin levels (from 13.2 ± 1.1 g/dL to 14.0 ± 1.1 g/dL, *P*<0.001) which was not observed in the placebo arm (13.5 ± 1.0 g/dL vs 13.5 ± 1.1 g/dL, *P*=0.95).

**Table 2:** Inflammation and iron status parameters at baseline and four weeks after the second vaccination.

	FCM (N=25)	Placebo (N=21)	P-value
Leucocyte count - 10^9^/L			
**Baseline**	7.0 ± 2.0	7.3 ± 1.6	0.28
**4 weeks after vaccination 2**	7.3 ± 1.9	7.1 ± 1.6	0.36
CRP, mg/L			
Baseline	1.6 (0.9 – 2.9)	1.2 (0.6 – 3.6)	0.50
**4 weeks after vaccination 2**	1.8 (0.8 – 5.4)	1.2 (0.7 – 2.3)	0.24
Iron, μg/dL			
Baseline	75.4 ± 25.7	78.2 ± 22.9	0.36
**4 weeks after vaccination 2**	98.8 ± 29.0	79.3 ± 34.1	0.02
Ferritin, μg/L			
Baseline	57 (29–79)	32 (24–62)	0.18
**4 weeks after vaccination 2**	457 (269 – 627)	41 (23 – 71)	<0.001
TSAT, %			
Baseline	21 ± 8	21 ± 8	0.49
**4 weeks after vaccination 2**	34 ± 12	21 ± 10	<0.001
TIBC, µg/dL			
Baseline	369 ± 70	378 ± 55	0.34
**4 weeks after vaccination 2**	303 ± 56	381 ± 52	<0.001

Inflammation and iron status parameters at baseline and four weeks after the second vaccination. Data are presented as mean ± standard deviation (SD), median with interquartile range (IQR) or number (n) with percentage (%). CRP, C-reactive protein; TIBC, total iron binding capacity; TSAT, transferrin saturation.

### Anti-SARS-CoV-2 antibody response

**Figure 3:**
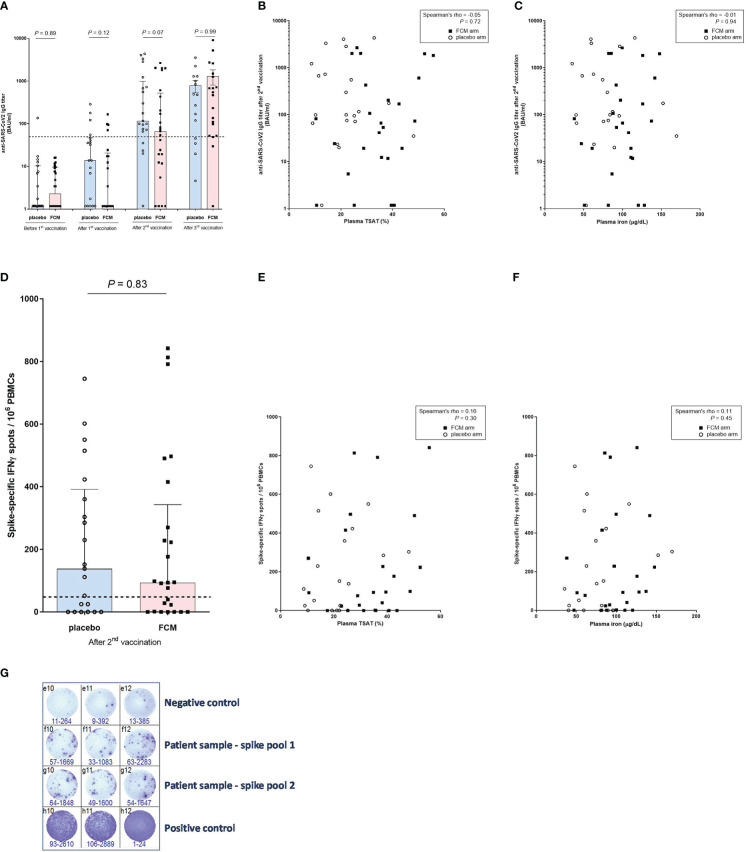
Anti-SARS-CoV-2 antibody and T-lymphocyte response. **(A)** Anti-SARS-CoV-2 IgG titers before vaccination and after the successive vaccinations in iron-deficient KTRs who had been treated with ferric carboxymaltose or placebo. The dashed horizontal line represents the threshold for IgG seropositivity. **(B)** Anti-SARS-CoV-2 IgG titers and transferrin saturation (in all participants). **(C)** Anti-SARS-CoV-2 IgG titers and plasma iron (in all participants). **(D)** SARS-CoV-specific T-lymphocyte response in iron-deficient KTRs who had been treated with ferric carboxymaltose or placebo. The dashed horizontal line represents the threshold for a positive T-lymphocyte response. **(E)** SARS-CoV-specific T-lymphocyte response and transferrin saturation (in all participants). **(F)** SARS-CoV-specific T-lymphocyte response and plasma iron (in all participants). **(G)** Example of representative results of an ELISpot assay, with which the SARS-CoV-2-specific T-lymphocyte response was measured in a patient (Spike pool 1 and 2). Interferon-gamma-producing T-lymphcytes after stimulation with SARS-CoV-2 Spike overlapping peptide pools are colored purple. All stimulations are performed in triplicate per peptide pool and the average of the six measurements is calculated. To correct for background activation, the average signal of the negative control is subtracted from the spike response.

There was no significant difference between the treatment groups in SARS-CoV-2-specific anti-RBD IgG concentration at four weeks after the second vaccination dose (*P*=0.07), which was one of the two co-primary outcomes of this study. Also after the first (*P*=0.12), or the third vaccination (P=0.99) there was no difference in SARS-CoV-2-specific anti-RBD IgG concentration between the study groups (**Figure 3A**). During the four weeks after the first vaccination, there was no significant increase in SARS-CoV-2-specific anti-RBD IgG concentration in patients in the FCM arm (2.31 [1.18–9.53] BAU/mL to 1.18 [1.18-20.63] BAU/mL, *P*=0.14). At four weeks after the second vaccination, SARS-CoV-2-specific anti-RBD IgG concentration significantly increased to 66.51 [12.02–517.59] BAU/mL in the FCM group (*P*<0.001 vs before first vaccination). Finally, four weeks after the third vaccination, SARS-CoV-2-specific anti-RBD IgG concentration further increased to 464.71 [52.13 – 1255.30] in the FCM group (*P*<0.001 vs before first vaccination). In the placebo arm, SARS-CoV-2-specific anti-RBD IgG concentration increased from 1.18 [1.18–10.33] BAU/mL before the first vaccination to 13.75 [1.18–46.18] BAU/mL four weeks later (*P*=0.03), to 115.97 [68.86–974.67] BAU/mL at four weeks after the second vaccination (*P*<0.001 vs before the first vaccination) and to 476.46 [45.00 – 1286.60] after the third vaccination (*P*=0.004 vs before first vaccination). An *a posteriori* power calculation indicated that, based on measured antibody levels, this study had 80% power to detect a 30% increase in (natural log-transformed) SARS-CoV-2-specific anti-RBD IgG concentrations at four weeks after the third vaccination.

After adjusting for total lymphocyte count (*P=*0.61) or eGFR (*P*=0.19) at baseline, there was still no significant difference in SARS-CoV-2-specific anti-RBD IgG concentration between the treatment groups at four weeks after the second vaccination. We found no correlation between TSAT (Spearman’s rho -0.05, *P*=0.72) or plasma ferritin (Spearman’s rho -0.15, *P*=0.33) or plasma iron (Spearman’s rho -0.01, *P*=0.94) and SARS-CoV-2-specific anti-RBD IgG concentration at four weeks after the second vaccination (**Figures 3B, C**). There was also no significant correlation between SARS-CoV-2-specific anti-RBD IgG concentration measured at four weeks after the second vaccination, and the total lymphocyte count at baseline (Spearman’s rho 0.26, *P*=0.09).

Seroconversion increased from 19% in the FCM group and 17% in the placebo group (*P*=0.85 between groups) at four weeks after the first vaccination to 56% in the FCM group and 80% in the placebo group (*P*=0.09 between groups) after the second vaccination and to 84% in the FCM group and 79% in the placebo group (*P*= 0.68 between groups) after the third vaccination. In all sensitivity analyses, results were highly comparable to those of the main analysis (**Supplementary Tables 1–24**).

### SARS-CoV-2-specific T-lymphocyte response

We subsequently evaluated the effect of FCM on the SARS-CoV2-specific cellular vaccination response, expressed in number of IFN-ɣ-producing T-lymphocytes after stimulation with SARS-CoV-2 Spike, at four weeks after the second vaccination. This was the second co-primary outcome of this study. KTRs in the FCM arm had a median of 93.3 [0.85–342.5] IFN-ɣ spots per 10^6^ PBMCs, compared to 138.3 [0.0–391.7] IFN-ɣ spots per 10^6^ PBMCs in the KTRs in the placebo arm (P=0.83, **Figure 3D**). Also after adjusting for total lymphocyte count (*P=*0.93) or eGFR (*P*=0.96) at baseline, there was no difference in SARS-CoV2-specific cellular response between the treatment groups after the second vaccination. The number of IFN-ɣ spots significantly correlated with the SARS-CoV-2-specific anti-RBD IgG concentration (Spearman’s rho 0.44, *P*=0.002), but not with TSAT (Spearman’s rho 0.16, *P*=0.30, [**Figure 3E**]), plasma ferritin (Spearman’s rho 0.00, *P*=0.98), plasma iron (Spearman’s rho 0.11, *P*=0.45, **Figure 3F**) or total lymphocyte count at baseline (Spearman’s rho 0.10, *P*=0.50). 60% of KTRs in the FCM group and 62% of KTRs in the placebo group were T-lymphocyte responders (P=0.90). The between-group differences were non-significant in all sensitivity analyses (**Supplementary Tables 25–36**). An example of the results of an ELISPOT assay is depicted in **Figure 3G**.

## Discussion

The main finding of this study is that correction of ID with FCM does not improve the humoral or cellular post-vaccination immune response against SARS-CoV-2 in KTRs. Although iron-deficient KTRs who were treated with FCM showed a significant increase in plasma ferritin and TSAT compared to placebo, there was no difference in SARS-CoV-2-specific anti-RBD IgG concentration, seroconversion rate or number of IFN-ɣ-producing T-lymphocytes after vaccination. These results are in contrast with studies in other populations, reporting improved vaccination efficacy after iron supplementation (15). A prior study showed that the antibody response after vaccination against measles virus was significantly stronger in iron-sufficient, compared to iron-deficient Chinese individuals (17). In Kenyan infants, higher TSAT predicted a stronger antibody response after vaccination against *Corynebacterium diphtheriae* and *Streptococcus pneumonia* while a lower transferrin receptor level was associated with a stronger antibody response after vaccination against poliovirus (18). In a randomized trial among the same population, oral iron supplementation before vaccination improved antibody response against measles virus (18). Iron is essential for activation, proliferation and function of T- and B-lymphocytes (17, 28), which might explain impaired vaccine efficacy associated with ID. Based on these findings we hypothesized that correction of ID would improve the immune response against SARS-CoV-2 after vaccination in KTRs. There are several potential explanations for the negative outcome of our study. First, other transplantation-related factors affecting the immune system, most importantly the use of immunosuppressive medication, may have overruled the potential effect of iron supplementation. The majority (83%) of participants was on triple immunosuppressive therapy and used mycophenolic acid; both are factors strongly associated with impaired vaccine response (10). The detrimental effect of mycophenolic acid on vaccine efficacy in KTRs was highlighted by a german study (29) reporting a beneficial effect of temporarily withholding antimetabolite treatment around the time of vaccination, although a recently published trial could not confirm these results (30). Second, we cannot exclude the possibility that iron supplementation has unfavorable effects on the immune system that might have counterbalanced potential beneficial effects. In a recently published meta-analysis, intravenous iron supplementation was associated with a higher infection risk (31), although this was not found in the PIVOTAL trial (32). While specific strains of pathogens need iron to thrive, intravenous iron supplementation may also indirectly increase the risk of infection by inducing oxidative stress, which is toxic to macrophages, neutrophils (33, 34), and lymphocytes (33, 35, 36). Third, the pathophysiological basis of ID in Dutch KTRs likely differs from ID in the previously reported populations, in which nutritional deficits and parasite infections may have played a major role (37). In our KTR population, ID is more likely induced by pro-inflammatory cytokines through upregulation of hepcidin, which prevents iron absorption from the gut and promotes iron entrapment in monocytes (38). In the context of inflammation, systemic ID may result from disordered iron distribution rather than an absolute deficit (39). It could be that only absolute ID affects vaccination response. However, in animal studies, it has been shown that not only absolute ID but also inflammation-associated ID affects T-lymphocyte response to vaccination against adenovirus (40). Furthermore, in humans with genetic hepcidin overexpression, antibody titers against various pathogens after vaccination are decreased (40). Finally, the definition of ID used in the current study may be too liberal. Since ferritin is an acute-phase protein, it can be increased by pro-inflammatory cytokines despite a depletion of iron (38). Therefore, we used a much higher cut-off value for plasma ferritin levels than what would be appropriate as a reference in the general population, although it is commonly used in chronic heart failure patients (41). In a sensitivity analysis including only KTRs with severe ID, although with limited statistical power, there was also no difference in SARS-CoV-2-specific vaccine efficacy between the two treatment arms (**Supplementary Tables 5, S16, S27**).

Notably, although there were no significant differences in antibody response between the treatment groups after any of the three vaccination doses, a small advance of the placebo-treated group seemed to decrease after each vaccination dose, until after the third dose, the median SARS-CoV-2-specific anti-RBD IgG concentration was slightly higher in the FCM-treated group. Another study (13), focusing on the lower antibody response to vaccination in individuals of higher age, showed that the difference between age groups decreased with each dose, thereby highlighting the efficacy of booster vaccination doses, which has also been observed in kidney transplant recipients (30).

In the current study, only 68% of patients had a positive SARS-CoV-2-specific anti-RBD IgG response and 61% had SARS-CoV-2-specific T-lymphocyte activation after the second vaccination. These numbers are similar to results of other studies among KTRs (6–10, 12). In accordance with prior studies (10, 12), there was a significant correlation between SARS-CoV-2-specific anti-RBD IgG titer and the number of IFN-ɣ spots per 10^6^ PBMCs. However, it should be emphasized that in some KTRs who remain seronegative after vaccination against SARS-CoV-2, a cellular antibody response can be detected (42). One patient in our study had a SARS-CoV-2-specific anti-RBD IgG concentration above the threshold for a positive response before the first vaccination. This patient was seronegative for total SARS-CoV-2-specific anti-RBD antibodies, but positive for antinucleocapsid antibodies. Therefore, it is unclear whether this patient had previous exposure to SARS-CoV-2 before or the positive IgG titer was based on cross-reactivity with antibodies against other coronaviruses or antigens. Previous studies among KTRs show an incidence of a positive pre-vaccination SARS-CoV-2-specific anti-RBD IgG response of 10% (12). Three patients had a total SARS-CoV-2- specific anti-RBD antibody OD above the threshold at baseline. Two of them also had anti-nucleocapsid antibodies, suggesting previous exposure to SARS-CoV-2. In a sensitivity analysis, all four patients were excluded, and this did not affect the results **(Supplementary Table 2, S13, S24**).

Our study has several strengths as well as limitations. We had the unique chance to assess vaccine efficacy within the scope of a running clinical trial assessing the impact of treatment with FCM versus placebo in iron-deficient KTRs. Another strength is the simultaneous availability of SARS-CoV-2-specific anti-RBD IgG concentration and data on SARS-CoV-2-specific T-lymphocyte activation. Limitations include the relatively small number of participants; nevertheless, since we did not find any trend towards a positive effect, a larger sample size would be unlikely to lead to a different outcome. The current study involved patients participating in an ongoing clinical trial who had not received all study treatments at the time of the Dutch national COVID-19 vaccination campaign, as well as patients who had finished their participation in the trial up to a year before vaccination. Therefore, there was considerable heterogenicity in the number of treatments received at the time of vaccination and the time between the last treatment and vaccination. Nevertheless, there was a clear difference in iron status between the two arms at the time of measurements four weeks after the second vaccination. At the time of the first vaccination, only a small amount of serum was collected from a finger capillary blood sample (collected with a fingerpick sampled at home). Unfortunately, these samples did not allow us to measure iron status parameters at that time. Furthermore, most patients received the mRNA-1273 vaccine whereas some received the mRNA-BNT162b2 vaccine. However, the results were robust in a sensitivity analysis excluding patients who were vaccinated with mRNA-BNT162b2 (**Supplementary Tables 4, S15, S26)**. We did not perform a pre-vaccination measurement of the SARS-CoV-2-specific T-lymphocyte activation. It can therefore not be excluded that some participants had baseline cellular reactivity, for example resulting from cross-reactivity against antigens of other coronaviruses, which is found in 11% of KTRs (12). Moreover, the results may be biased by other differences between the treatment arms. Patients in both groups were generally well balanced at baseline except for a slightly but significantly lower lymphocyte count and eGFR in the FCM arm. Although lymphocytopenia might restrain an adequate immune response after vaccination (10), thereby masking an effect of FCM, we did not observe an effect of FCM on vaccine efficacy after adjusting for these potential confounders. Of note, there was no correlation between lymphocyte count and number of IFN-ɣ-producing T-lymphocytes or antibody titer. Furthermore, we have not measured neutralizing antibody responses, which would have been an interesting secondary outcome. Finally, the results of our study may be specific for KTRs and cannot be extrapolated to other immunocompromised populations.

In conclusion, in KTRs with ID, intravenous iron supplementation efficiently restored iron status but did not improve the humoral or cellular immune response against SARS-CoV-2 after three vaccinations.

## Data availability statement

The raw data supporting the conclusions of this article will be made available by the authors, without undue reservation.

## Ethics statement

The studies involving human participants were reviewed and approved by Medical ethical committee of the University Medical Center Groningen (METc 2018/482). The patients/participants provided their written informed consent to participate in this study.

## Author contributions

MD and JV developed the study design. JV coordinated the trial. MHdB supervised the trial. DB, MS, TR and J-SS contributed to the study design. DA, RJ, TN, MvdH.and MS performed analyses. All authors contributed to the article and approved the submitted version.
