# Supplementary material for: Ferric carboxymaltose and SARS-CoV-2 vaccination-induced immunogenicity in kidney transplant recipients with iron deficiency: The COVAC-EFFECT randomized controlled trial
Source: Front Immunol. 2023 Jan 4;13:1017178. doi: 10.3389/fimmu.2022.1017178 (PMC9822258; doi:10.3389/fimmu.2022.1017178)
Supplement: Supplementary file 1 [file DataSheet_1.pdf]

Supplemental Material

Supplemental Tables

Supplemental Tables 1-12: SARS-CoV-2-specific anti-RBD IgG titer before and after vaccination against SARS-CoV-2 in FCM versus placebo arm in different sensitivity analyses

Supplemental Tables 13-24: Seroconversion rate of SARS-CoV-2-specific anti-RBD IgG antibodies after the first, the second and the third vaccination against SARS-CoV-2 in the FCM arm versus the placebo arm in different sensitivity analyses

Supplemental Tables 25-36: SARS-CoV-2-specific T-lymphocyte response before and after the second vaccination against SARS-CoV-2 in FCM versus placebo arm in different sensitivity analyses

Supplemental Table 1. SARS-CoV-2-specific anti-RBD IgG titer before and after vaccination against SARS-CoV-2 in FCM versus placebo arm in a per-protocol analysis excluding patients with iron deficiency in the FCM arm or without iron deficiency in the placebo arm at four weeks after second vaccination

SARS-CoV-2-specific anti-RBD IgG titer (BAU/mL)	**FCM** N=23	**Placebo** N=20	**P-value (FCM vs placebo)**
**Before vaccination 1**	3.44 [1.18 – 9.81]	1.47 [1.18 – 10.89]	
**4 weeks after vaccination 1**	2.31 [1.18 – 22.36]	23.03 [1.61 – 48.41]*	0.11
**4 weeks after vaccination 2**	66.51 [12.34 – 603.95]*	107.75 [67.33 – 1098.33]*	0.09
**4 weeks after vaccination 3**	541.68 [74.73 – 1374.35]*	476.46 [45.00 – 1286.60]*	0.82

**P*<0.05 vs. before vaccination 1

Supplemental Table 2. SARS-CoV-2-specific anti-RBD IgG titer before and after vaccination against SARS-CoV-2 in FCM versus placebo arm in a sensitivity analysis excluding patients who were seropositive for total SARS-CoV-2-specific anti-RBD antibodies at baseline

SARS-CoV-2-specific anti-RBD IgG titer (BAU/mL)	**FCM** N=23	**Placebo** N=19	**P-value (FCM vs placebo)**
**Before vaccination 1**	2.31 [1.18 – 9.81]	1.18 [1.18 – 8.96]	
**4 weeks after vaccination 1**	1.18 [1.18 – 22.36]	13.75 [1.18 – 38.25]*	0.26
**4 weeks after vaccination 2**	53.70 [11.70 – 203.28]*	115.97 [67.80 – 727.33]*	0.06
**4 weeks after vaccination 3**	464.71 [74.73 – 986.44]*	476.46 [82.19 – 1143.27]*	0.87

**P*<0.05 vs. before vaccination 1

Supplemental Table 3. SARS-CoV-2-specific anti-RBD IgG titer before and after vaccination against SARS-CoV-2 in FCM versus placebo arm in a sensitivity analysis excluding patients who were IgG deficient at baseline

SARS-CoV-2-specific anti-RBD IgG titer (BAU/mL)	**FCM** N=18	**Placebo** N=17	**P-value (FCM vs placebo)**
**Before vaccination 1**	5.17 [1.18 – 10.67]	3.44 [1.18 – 12.57]	
**4 weeks after vaccination 1**	6.20 [1.18 – 22.36]	13.75 [1.18 – 46.18]	0.34
**4 weeks after vaccination 2**	77.55 [17.54 – 908.28]*	115.97 [68.86 – 700.89]*	0.27
**4 weeks after vaccination 3**	610.12 [52.11 – 1771.20]*	168.61 [34.31 – 837.78]*	0.18

**P*<0.05 vs. before vaccination 1

Supplemental Table 4. SARS-CoV-2-specific anti-RBD IgG titer before and after vaccination against SARS-CoV-2 in FCM versus placebo arm in a sensitivity analysis excluding patients who had received the mRNA-BNT16B2 vaccine

SARS-CoV-2-specific anti-RBD IgG titer (BAU/mL)	**FCM**<br>N=23	**Placebo**<br>N=18	**P-value**<br>**(FCM vs placebo)**
**Before vaccination 1**	3.44 [1.18 – 9.64]	1.47 [1.18 – 9.64]	
**4 weeks after vaccination 1**	1.18 [1.18 – 17.16]	32.31 [1.18 – 50.65]*	0.14
**4 weeks after vaccination 2**	73.2 [19.3 – 603.95]*	145.53 [58.13 – 1630.31]*	0.15
**4 weeks after vaccination 3**	464.71 [74.77 – 1374.35]*	378.65 [36.98 – 1289.53]*	0.74

**P*<0.05 vs. before vaccination 1

Supplemental Table 5. SARS-CoV-2-specific anti-RBD IgG titer before and after vaccination against SARS-CoV-2 in FCM versus placebo arm in a sensitivity analysis including only patients with severe iron deficiency at baseline

SARS-CoV-2-specific anti-RBD IgG titer (BAU/mL)	**FCM**<br>N=7	**Placebo**<br>N=10	**P-value**<br>**(FCM vs placebo)**
**Before vaccination 1**	1.18 [1.18 – 9.18]	1.47 [1.18 – 6.03]	
**4 weeks after vaccination 1**	1.18 [1.17 – 49.36]	35.6 [1.47 – 47.89]	0.15
**4 weeks after vaccination 2**	66.51 [1.18 – 431.23]*	87.36 [32.19 – 1747.81]*	0.23
**4 weeks after vaccination 3**	116.56 [52.11 – 1840.95]*	269.66 [45.00 – 1290.50]*	1.00

*P<0.05 vs. before vaccination 1

Supplemental Table 6. SARS-CoV-2-specific anti-RBD IgG titer before and after vaccination against SARS-CoV-2 in FCM versus placebo arm in a sensitivity analysis including only patients on dual immunosuppressive therapy

SARS-CoV-2-specific anti-RBD IgG titer (BAU/mL)	**FCM**<br>N=4	**Placebo**<br>N=4	**P-value**<br>**(FCM vs placebo)**
**Before vaccination 1**	3.44 [1.18 – 3.44]	1.18 [1.18 – 13.17]	
**4 weeks after vaccination 1**	35.84 [1.16 – 88.66]	11.47 [7.46 – 38.07]	1.00
**4 weeks after vaccination 2**	947.23 [19.19 – 1953.44]	199.29 [42.27 – 620.41]	0.89
**4 weeks after vaccination 3**	359.38 [1.18 – 359.38]	478.58 [44.65 – 959.39]	0.53

*P<0.05 vs. before vaccination 1

Supplemental Table 7. SARS-CoV-2-specific anti-RBD IgG titer before and after vaccination against SARS-CoV-2 in FCM versus placebo arm in a sensitivity analysis including only patients on triple immunosuppressive therapy

SARS-CoV-2-specific anti-RBD IgG titer (BAU/mL)	**FCM** N=21	**Placebo** N=17	**P-value (FCM vs placebo)**
**Before vaccination 1**	1.18 [1.18 – 9.41]	1.75 [1.18 – 10.33]	
**4 weeks after vaccination 1**	1.18 [1.18 – 17.16]	32.31 [1.18 – 55.11]	0.12
**4 weeks after vaccination 2**	53.70 [12.02 – 317.26]*	115.97 [68.86 – 2038.63]*	0.05
**4 weeks after vaccination 3**	464.71 [74.77 – 1374.35]*	476.46 [45.00 – 1290.50]*	0.93

**P*<0.05 vs. before vaccination 1

Supplemental Table 8. SARS-CoV-2-specific anti-RBD IgG titer before and after vaccination against SARS-CoV-2 in FCM versus placebo arm in a sensitivity analysis excluding patients who had received anti-thymocyte globulin, methylprednisolone or alemtuzumab during the two years before vaccination

SARS-CoV-2-specific anti-RBD IgG titer (BAU/mL)	**FCM** N=24	**Placebo** N=21	**P-value (FCM vs placebo)**
**Before vaccination 1**	1.18 [1.18 – 9.64]	1.18 [1.18 – 10.33]	
**4 weeks after vaccination 1**	2.31 [1.18 – 22.36]	13.75 [1.18 – 46.18]*	0.15
**4 weeks after vaccination 2**	69.86 [13.59 – 560.77]*	115.97 [68.86 – 974.67]*	0.10
**4 weeks after vaccination 3**	503.20 [86.09 – 1314.83]*	476.46 [45.00 – 1286.60]*	0.90

**P*<0.05 vs. before vaccination 1

Supplemental Table 9. SARS-CoV-2-specific anti-RBD IgG titer before and after vaccination against SARS-CoV-2 in FCM versus placebo arm in a sensitivity analysis in patients using mycophenolic acid

SARS-CoV-2-specific anti-RBD IgG titer (BAU/mL)	**FCM** N=20	**Placebo** N=18	**P-value (FCM vs placebo)**
**Before vaccination 1**	1.18 [1.18 – 9.18]	1.47 [1.18 – 9.64]	
**4 weeks after vaccination 1**	1.18 [1.18 – 15.96]	23.03 [1.18 – 51.50]	0.09
**4 weeks after vaccination 2**	63.45 [14.07 – 374.24]*	107.75 [70.38 – 1630.31]*	0.08
**4 weeks after vaccination 3**	464.71 [52.09 – 1374.35]*	482.05 [75.90 – 1289.53]*	0.88

**P*<0.05 vs. before vaccination 1

Supplemental Table 10. SARS-CoV-2-specific anti-RBD IgG titer before and after vaccination against SARS-CoV-2 in FCM versus placebo arm in a sensitivity analysis including only participants using 500mg mycophenolic acid twice daily

SARS-CoV-2-specific anti-RBD IgG titer (BAU/mL)	**FCM**<br>N=15	**Placebo**<br>N=13	**P-value**<br>**(FCM vs placebo)**
**Before vaccination 1**	1.18 [1.18 – 7.06]	1.18 [1.18 – 6.55]	
**4 weeks after vaccination 1**	1.18 [1.18 – 11.49]	6.89 [1.18 – 40.66]*	0.26
**4 weeks after vaccination 2**	81.90 [19.27 – 431.23]*	175.08 [73.56 – 2038.63]*	0.08
**4 weeks after vaccination 3**	464.71 [80.88 – 1374.35]*	285.00 [25.00 – 469.15]*	0.21

*P<0.05 vs. before vaccination 1

Supplemental Table 11. SARS-CoV-2-specific anti-RBD IgG titer before and after vaccination against SARS-CoV-2 in FCM versus placebo arm in a sensitivity analysis including only men

SARS-CoV-2-specific anti-RBD IgG titer (BAU/mL)	**FCM**<br>N=24	**Placebo**<br>N=21	**P-value**<br>**(FCM vs placebo)**
**Before vaccination 1**	4.82 [1.18 – 10.33]	1.18 [1.18 – 8.27]	
**4 weeks after vaccination 1**	10.65 [1.18 – 42.45]	33.96 [5.46 – 48.41]*	0.31
**4 weeks after vaccination 2**	60.11 [8.95 – 1657.93]*	145.53 [67.33 – 2446.94]*	0.19
**4 weeks after vaccination 3**	607.48 [85.16 – 2630.55]*	837.78 [45.00 – 1290.50]*	0.92

*P<0.05 vs. before vaccination 1

Supplemental Table 12. SARS-CoV-2-specific anti-RBD IgG titer before and after vaccination against SARS-CoV-2 in FCM versus placebo arm in a sensitivity analysis including only women

SARS-CoV-2-specific anti-RBD IgG titer (BAU/mL)	**FCM**<br>N=13	**Placebo**<br>N=5	**P-value**<br>**(FCM vs placebo)**
**Before vaccination 1**	1.18 [1.18 – 9.41]	6.89 [1.47 – 76.73]	
**4 weeks after vaccination 1**	1.18 [1.18 – 17.16]	1.75 [1.18 – 67.98]	0.38
**4 weeks after vaccination 2**	73.20 [12.02 – 317.26]*	99.52 [64.98 – 487.04]	0.34
**4 weeks after vaccination 3**	135.71 [52.09 – 698.07]*	144.00 [33.28 – 399.50]	0.71

*P<0.05 vs. before vaccination 1

Supplemental Table 13. Seroconversion rate of SARS-CoV-2-specific anti-RBD IgG antibodies after the first, the second and the third vaccination against SARS-CoV-2 in FCM versus placebo arm in a per-protocol analysis excluding patients with iron deficiency in the FCM arm or without iron deficiency in the placebo arm at four weeks after second vaccination

Seroconversion rate, n (%)	**FCM**<br>N=23	**Placebo**<br>N=20	**P-value**<br>**(FCM vs placebo)**
**4 weeks after vaccination 1**	4 (20)	3 (18)	0.86
**4 weeks after vaccination 2**	13 (57)	15 (79)	0.13
**4 weeks after vaccination 3**	14 (82)	11 (79)	0.79

Supplemental Table 14. Seroconversion of SARS-CoV-2-specific anti-RBD IgG antibodies after the first, the second and the third vaccination against SARS-CoV-2 in the FCM arm versus the placebo arm in a sensitivity analysis excluding patients who were seropositive for total SARS-CoV-2-specific anti-RBD antibodies at baseline

Seroconversion rate, n (%)	**FCM**<br>N=23	**Placebo**<br>N=19	**P-value**<br>**(FCM vs placebo)**
**4 weeks after vaccination 1**	4 (20)	2 (12)	0.50
**4 weeks after vaccination 2**	12 (52)	15 (79)	0.07
**4 weeks after vaccination 3**	14 (82)	10 (77)	0.71

Supplemental Table 15. Seroconversion of SARS-CoV-2-specific anti-RBD IgG antibodies after the first, the second and the third vaccination against SARS-CoV-2 in the FCM arm versus the placebo arm in a sensitivity analysis excluding patients who were IgG deficient at baseline

Seroconversion rate, n (%)	**FCM**<br>N=18	**Placebo**<br>N=17	**P-value**<br>**(FCM vs placebo)**
**4 weeks after vaccination 1**	3 (19)	2 (14)	0.74
**4 weeks after vaccination 2**	11 (61)	13 (81)	0.20
**4 weeks after vaccination 3**	12 (86)	7 (70)	0.35

Supplemental Table 16. Seroconversion of SARS-CoV-2-specific anti-RBD IgG antibodies after the first, the second and the third vaccination against SARS-CoV-2 in the FCM arm versus the placebo arm in a sensitivity analysis excluding patients who had received the mRNA-BNT16B2 vaccination

Seroconversion rate, n (%)	**FCM**<br>N=23	**Placebo**<br>N=18	**P-value**<br>**(FCM vs placebo)**
**4 weeks after vaccination 1**	4 (21)	3 (19)	0.87
**4 weeks after vaccination 2**	14 (61)	13 (76)	0.30
**4 weeks after vaccination 3**	15 (88)	8 (73)	0.30

Supplemental Table 17. Seroconversion of SARS-CoV-2-specific anti-RBD IgG antibodies after the first, the second and the third vaccination against SARS-CoV-2 in the FCM arm versus the placebo arm in a sensitivity analysis including only patients with severe iron deficiency at baseline

Seroconversion rate, n (%)	**FCM**<br>N=7	**Placebo**<br>N=10	**P-value**<br>**(FCM vs placebo)**
**4 weeks after vaccination 1**	1 (20)	2 (22)	0.92
**4 weeks after vaccination 2**	4 (57)	7 (70)	0.59
**4 weeks after vaccination 3**	6 (100)	5 (71)	0.16

Supplemental Table 18. Seroconversion of SARS-CoV-2-specific anti-RBD IgG antibodies after the first, the second and the third vaccination against SARS-CoV-2 in the FCM arm versus the placebo arm in a sensitivity analysis including patients on dual immunosuppressive therapy

Seroconversion rate, n (%)	**FCM**<br>N=4	**Placebo**<br>N=4	**P-value**<br>**(FCM vs placebo)**
**4 weeks after vaccination 1**	2 (50)	0 (0)	0.10
**4 weeks after vaccination 2**	3 (75)	3 (75)	1.0
**4 weeks after vaccination 3**	1 (50)	3 (75)	0.54

Supplemental Table 19. Seroconversion of SARS-CoV-2-specific anti-RBD IgG antibodies after the first, the second and the third vaccination against SARS-CoV-2 in the FCM arm versus the placebo arm in a sensitivity analysis including patients on triple immunosuppressive therapy

Seroconversion rate, n (%)	**FCM**<br>N=21	**Placebo**<br>N=17	**P-value**<br>**(FCM vs placebo)**
**4 weeks after vaccination 1**	2 (12)	3 (21)	0.47
**4 weeks after vaccination 2**	11 (52)	13 (81)	0.07
**4 weeks after vaccination 3**	15 (88)	8 (80)	0.56

Supplemental Table 20. Seroconversion of SARS-CoV-2-specific anti-RBD IgG antibodies after the first, the second and the third vaccination against SARS-CoV-2 in the FCM arm versus the placebo arm in a sensitivity analysis excluding patients who had received anti-thymocyte globulin, methylprednisolone or alemtuzumab during the two years before vaccination

Seroconversion rate, n (%)	**FCM**<br>N=24	**Placebo**<br>N=21	**P-value**<br>**(FCM vs placebo)**
**4 weeks after vaccination 1**	4 (20)	3 (17)	0.79
**4 weeks after vaccination 2**	14 (58)	16 (80)	0.12
**4 weeks after vaccination 3**	15 (83)	11 (79)	0.73

Supplemental Table 21. Seroconversion of SARS-CoV-2-specific anti-RBD IgG antibodies after the first, the second and the third vaccination against SARS-CoV-2 in the FCM arm versus the placebo arm in a sensitivity analysis in patients using mycophenolic acid

Seroconversion rate, n (%)	**FCM**<br>N=20	**Placebo**<br>N=18	**P-value**<br>**(FCM vs placebo)**
**4 weeks after vaccination 1**	2 (13)	3 (20)	0.57
**4 weeks after vaccination 2**	11 (55)	14 (82)	0.08
**4 weeks after vaccination 3**	14 (82)	9 (82)	0.97

Supplemental Table 22. Seroconversion of SARS-CoV-2-specific anti-RBD IgG antibodies after the first, the second and the third vaccination against SARS-CoV-2 in the FCM arm versus the placebo arm in a sensitivity analysis in patients using 500mg mycophenolic acid twice daily

Seroconversion rate, n (%)	**FCM**<br>N=15	**Placebo**<br>N=13	**P-value**<br>**(FCM vs placebo)**
**4 weeks after vaccination 1**	1 (8))	1 (18)	0.48
**4 weeks after vaccination 2**	9 (60)	12 (92)	0.05
**4 weeks after vaccination 3**	11 (85)	7 (100)	0.27

Supplemental Table 23. Seroconversion of SARS-CoV-2-specific anti-RBD IgG antibodies after the first, the second and the third vaccination against SARS-CoV-2 in the FCM arm versus the placebo arm in a sensitivity analysis including only men

Seroconversion rate, n (%)	**FCM**<br>N=12	**Placebo**<br>N=16	**P-value**<br>**(FCM vs placebo)**
**4 weeks after vaccination 1**	2 (20)	3 (21)	0.93
**4 weeks after vaccination 2**	7 (58)	13 (81)	0.18
**4 weeks after vaccination 3**	8 (80)	8 (73)	0.70

Supplemental Table 24. Seroconversion of SARS-CoV-2-specific anti-RBD IgG antibodies after the first, the second and the third vaccination against SARS-CoV-2 in the FCM arm versus the placebo arm in a sensitivity analysis including only women

Seroconversion rate, n (%)	**FCM**<br>N=13	**Placebo**<br>N=5	**P-value**<br>**(FCM vs placebo)**
**4 weeks after vaccination 1**	2 (18)	0 (0)	0.36
**4 weeks after vaccination 2**	7 (54)	3 (75)	0.45
**4 weeks after vaccination 3**	8 (89)	3 (100)	0.55

Supplemental Table 25. SARS-CoV-2-specific T-lymphocyte response before and after vaccination against SARS-CoV-2 in FCM versus placebo arm in a per-protocol analysis excluding patients with iron deficiency in the FCM arm or without iron deficiency in the placebo arm at four weeks after second vaccination

SARS-CoV2-specific cellular response (IFN-ɣ spots per 10^6 PBMCs)	**FCM** N=23	**Placebo** N=20	**P-value (FCM vs placebo)**
**4 weeks after vaccination 2**	95.0 [0.0 – 415.0]	125.0 [0.0 – 407.5]	1.0

Supplemental Table 26. SARS-CoV-2-specific T-lymphocyte response before and after vaccination against SARS-CoV-2 in the FCM arm versus the placebo arm in a sensitivity analysis excluding patients who were seropositive for total SARS-CoV-2-specific anti-RBD antibodies at baseline

SARS-CoV2-specific cellular response (IFN-ɣ spots per 10^6 PBMCs)	**FCM** N=23	**Placebo** N=20	**P-value (FCM vs placebo)**
**4 weeks after vaccination 2**	93.3 [0.0 – 270.0]	138.3 [0.0 – 360.0]	0.67

Supplemental Table 27. SARS-CoV-2-specific T-lymphocyte response before and after vaccination against SARS-CoV-2 in the FCM arm versus the placebo arm in a sensitivity analysis excluding patients who were IgG deficient at baseline

SARS-CoV2-specific cellular response (IFN-ɣ spots per 10^6 PBMCs)	**FCM** N=18	**Placebo** N=17	**P-value (FCM vs placebo)**
**4 weeks after vaccination 2**	96.7 [17.5 – 491.7]	151.7 [12.5 – 469.2]	1.0

Supplemental Table 28. SARS-CoV-2-specific T-lymphocyte response before and after vaccination against SARS-CoV-2 in FCM versus placebo arm in a sensitivity analysis excluding patients who had received the mRNA-BNT16B2 vaccination

SARS-CoV2-specific cellular response (IFN-ɣ spots per 10^6 PBMCs)	**FCM** N=23	**Placebo** N=18	**P-value (FCM vs placebo)**
**4 weeks after vaccination 2**	95.0 [1.7 – 415.0]	190.9 [18.8 – 446.2]	0.58

Supplemental Table 29. SARS-CoV-2-specific T-lymphocyte response before and after vaccination against SARS-CoV-2 in the FCM arm versus the placebo arm in a sensitivity analysis including only patients with severe iron deficiency at baseline

SARS-CoV2-specific cellular response (IFN-ɣ spots per 10^6 PBMCs)	**FCM** N=7	**Placebo** N=10	**P-value (FCM vs placebo)**
**4 weeks after vaccination 2**	28.3 [0.0 – 98.3]	227.5 [25.0 – 562.9]	0.09

Supplemental Table 30. SARS-CoV-2-specific T-lymphocyte response before and after vaccination against SARS-CoV-2 in the FCM arm versus the placebo arm in a sensitivity analysis including only patients on dual immunosuppressive therapy

SARS-CoV2-specific cellular response (IFN-ɣ spots per 10^6 PBMCs)	**FCM** N=4	**Placebo** N=4	**P-value (FCM vs placebo)**
**4 weeks after vaccination 2**	160.8 [24.6 – 687.1]	115.0 [0.0 – 508.8]	0.89

Supplemental Table 31. SARS-CoV-2-specific T-lymphocyte response before and after vaccination against SARS-CoV-2 in the FCM arm versus the placebo arm in a sensitivity analysis including only patients on triple immunosuppressive therapy

SARS-CoV2-specific cellular response (IFN-ɣ spots per 10^6 PBMCs)	**FCM** N=21	**Placebo** N=17	**P-value (FCM vs placebo)**
**4 weeks after vaccination 2**	91.7 [0.9 – 342.5]	138.3 [12.5 – 391.7]	0.66

Supplemental Table 32. SARS-CoV-2-specific T-lymphocyte response before and after vaccination against SARS-CoV-2 in the FCM arm versus the placebo arm in a sensitivity analysis excluding patients who had received anti-thymocyte globulin, methylprednisolone or alemtuzumab during the two years before vaccination

SARS-CoV2-specific cellular response (IFN-ɣ spots per 10^6 PBMCs)	**FCM** N=24	**Placebo** N=21	**P-value (FCM vs placebo)**
**4 weeks after vaccination 2**	94.2 [7.1 – 378.8]	138.3 [0.0 – 391.7]	0.96

Supplemental Table 33. SARS-CoV-2-specific T-lymphocyte response before and after vaccination against SARS-CoV-2 in FCM versus placebo arm in a sensitivity analysis in patients using mycophenolic acid

SARS-CoV2-specific cellular response (IFN-ɣ spots per 10^6 PBMCs)	**FCM**	**Placebo** N=18	**P-value (FCM vs placebo)**
**4 weeks after vaccination 2**	94.2 [0.0 – 378.8]	125.0 [0.0 – 375.8]	0.90

Supplemental Table 34. SARS-CoV-2-specific T-lymphocyte response before and after vaccination against SARS-CoV-2 in FCM versus placebo arm in a sensitivity analysis in patients using 500mg mycophenolic acid twice daily

SARS-CoV2-specific cellular response (IFN-ɣ spots per 10^6 PBMCs)	**FCM** N=15	**Placebo** N=13	**P-value (FCM vs placebo)**
**4 weeks after vaccination 2**	95.0 [28.3 – 490.0]	285.0 [25.0 – 469.2]	0.65

Supplemental Table 35. SARS-CoV-2-specific T-lymphocyte response before and after vaccination against SARS-CoV-2 in the FCM arm versus the placebo arm in a sensitivity analysis including only men

SARS-CoV2-specific cellular response (IFN-ɣ spots per 10^6 PBMCs)	**FCM** N=12	**Placebo** N=16	**P-value (FCM vs placebo)**
**4 weeks after vaccination 2**	135.0 [0.4 – 495.0]	125.0 [6.3 – 407.5]	0.87

Supplemental Table 36. SARS-CoV-2-specific T-lymphocyte response before and after vaccination against SARS-CoV-2 in the FCM arm versus the placebo arm in a sensitivity analysis including only women

SARS-CoV2-specific cellular response (IFN-ɣ spots per 10^6 PBMCs)	**FCM** N=13	**Placebo** N=5	**P-value (FCM vs placebo)**
**4 weeks after vaccination 2**	91.7 [14.2 – 225.8]	151.7 [0.0 – 524.2]	0.78
